# Are first-trimester pregnant women consuming adequate and diverse diet? A hospital-based cross-sectional study in Karachi, Pakistan

**DOI:** 10.1186/s40795-024-00912-3

**Published:** 2024-07-24

**Authors:** Khadija Vadsaria, Nuruddin Mohammed, Shelina Aamir, Rozina Nuruddin

**Affiliations:** 1 Medical College, Aga Khan University, Stadium Road, Karachi, 74800 Pakistan; 2 Department of Obstetrics and Gynaecology, Aga Khan University Hospital, Stadium Road, Karachi, 74800 Pakistan; 3 Psychcare, F-67/1, 3rd Avenue, Block 5, Clifton, Karachi, 75600 Pakistan; 4 Department of Community Health Sciences, Aga Khan University, Stadium Road, PO Box 3500, Karachi, 74800 Pakistan

**Keywords:** Dietary adequacy, Dietary risk score, First trimester, Pakistan, Pregnancy

## Abstract

**Background:**

Maintaining a healthy and diverse diet during pregnancy is crucial for maternal well-being and fetal development. The first trimester marks the beginning of vital developmental processes influenced by maternal nutritional status. Therefore, we aimed to determine dietary adequacy and diversity among first-trimester pregnant women.

**Methods:**

In this cross-sectional study, we recruited 306 first-trimester pregnant women from the antenatal clinics of Aga Khan University Hospital, Karachi (January 2020 to September 2021). Eligible women possessed smartphones (for the mHealth intervention trial) and reported no major comorbidities or medication use. Data about socio-demographic, obstetric, and dietary history were collected through interviews using a structured questionnaire. Booking weight, height, blood pressure, and haemoglobin levels were extracted from medical records. An aggregate dietary risk score (DRS) was calculated separately for quantity and quality by summing the DRS for each of the six major food groups. A score of 0 was assigned to adequate, 1.5 to intermediate, and 3 to inadequate quantity or quality categories. Data were analysed using STATA 14.0.

**Results:**

The mean ± SD for DRS quantity and quality were 10.6 ± 2.4 and 7.5 ± 2.5, respectively. Adequate dietary quantity and quality per week for starch-based food were reported by 14.4% and 21.2%, for vegetables by 0.3% and 49%, for fruits by 41.2% and 88.6%, for animal and plant protein by 19% and 0%, for milk and milk products by 1% and 37.6% and for oils and fats by 90.5% and 8.8%, respectively. Sweet and savoury snacks were eaten by 74.8% and 53.9%, respectively. Ready-made meals, carbonated beverages, packaged juices, and additional salt were consumed by 55.2%, 46.4%, 34.3%, and 7.5%, respectively. The median (IQR) water intake was 6 (4–8) glasses/day.

**Conclusions:**

During the early stages of pregnancy, women enrolled for antenatal care at an urban private tertiary care hospital report inadequate dietary intake for various food groups, except for the quantity of oils/fats and the quality of fruit consumption. Poor dietary practices underscore the need for focused and impactful dietary counselling during the initial stages of pregnancy.

**Supplementary Information:**

The online version contains supplementary material available at 10.1186/s40795-024-00912-3.

## Background

Pregnancy marks the critical period of accelerated physiological changes from conception until birth [1]. The first trimester is particularly significant, involving placental development, organogenesis, and maternal endocrine and metabolic systems’ adaptation for the subsequent trimesters. For clinicians and health care practitioners, it is the most accessible period for identifying suboptimal lifestyle behaviours, with the opportunity for early intervention [2].

One such behaviour is the dietary choices during pregnancy that significantly influence the in-utero environment, fetal metabolic programming, and growth trajectories. In particular, dietary intake during the first trimester may serve as a harbinger of dietary patterns throughout pregnancy. Adequate dietary intake involves both quantity and quality, ensuring sufficient intake of macronutrients and micronutrients and a variety of food choices to meet biological needs [3–5].

An adequate diet during pregnancy has been linked to favourable pregnancy outcomes [6, 7]. A diet rich in fruits and dairy products significantly reduces the risk of gestational diabetes mellitus (GDM) (aOR: 0.50, CI: 0.284–0.882) [8]. Additionally, eating fish, poultry, dairy products, unrefined grains, fruits, vegetables, legumes, and oils lowers the risk of preterm birth (RR: 0.10, CI: 0.01, 0.77) [9].

Conversely, a Western diet high in red and processed meat, processed grains, sugar, and fried items increases the risk of GDM (RR: 1.63, 95% CI: 1.20–2.21) [10]. Similarly, low adherence to the Mediterranean diet, comprising vegetables, fish, pulses, legumes, and vegetable oils, raises the risk of high systolic blood pressure early in pregnancy (β: 1.1, 95% CI: 0.2–2.2). This risk intensifies with high adherence to a traditional diet, including red meat and potatoes (β: 1.8, 95% CI: 0.7–2.9) [11]. Furthermore, inadequate dietary diversity is associated with an increased risk of low birth weight (LBW) (aRR: 6.4, 95% CI: 3.4, 12) and preterm birth (aRR: 6.3, 95% CI: 3.3, 11.95) [12].

Despite evidence suggesting the importance of a well-balanced diet during pregnancy, many women in low- and middle-income countries (LMICs), particularly in South Asia, face poor nutrition. Their diet often consists of staple foods, lacks diversity, and is nutritionally deficient [5, 13]. As these regions experience nutritional transition, demonstrated by a shift towards unhealthy dietary choices, a triple burden of malnutrition emerges.

A similar situation is observed in Pakistan, where a notable proportion of women of reproductive age (WRA) are underweight (14.5%), overweight (24%), or obese (14%) and only 27.6% meet the minimum dietary diversity. These nutritional indicators differ by the area of residence, with rural women (R) more underweight (R: 16% vs. U: 12.1%) and urban women (U) more overweight (R: 17.3% vs. U: 26.8%) or obese (R: 11.8% vs. U: 22.7%). Sindh Province, in particular, exhibits worse nutritional indicators, with 22.8% of women being underweight and only 16.5% achieving the minimum dietary diversity [14]. These conditions perpetuate an intergenerational cycle of malnutrition when undernourished women become pregnant.

In Pakistan, limited small-scale studies have examined dietary intake during pregnancy. A study conducted in Faisalabad revealed that 67.5% of pregnant women consumed balanced diets [15] and in Islamabad 89% achieved medium-level dietary diversity (scores of 5–7 out of 10) [16]. However, none of these studies explicitly studied the quantity and quality of each food group during the first trimester.

Addressing malnutrition, crucial for sustainable development goals (SDGs), demands a comprehensive approach. Many SDGs are intricately connected to the developmental origin of health and disease, emphasising how experiences during the first 1000 days profoundly shape future health risks [17].

Optimal nutrition from preconception is vital for ensuring a positive pregnancy experience. However, in Pakistan, where preconception care is uncommon, the first trimester becomes the closest point of contact to address dietary inadequacies through counselling. To our knowledge, limited studies have examined the dietary intake of pregnant women with minimal emphasis on the first trimester.

Therefore, in this study, we aimed to determine the dietary adequacy (quantity-consumption of adequate portions) and diversity (quality-inclusion of a variety of food items) among first-trimester pregnant women visiting a tertiary care hospital in Karachi.

## Methods

### Study design, setting, and population

A hospital-based cross-sectional study was conducted from January 13, 2020, to September 30, 2021, at the Aga Khan University Hospital (AKUH) in Karachi, Pakistan [18]. As the provincial capital of Sindh and the largest metropolis in Pakistan, Karachi is the commercial hub that accommodates migrants from across the country. AKUH is a private, not-for-profit tertiary care institution that is Joint Commission International Accredited and provides a comprehensive array of medical services to residents of Karachi and people from different parts of the country [19]. The hospital has various specialities, with trained and competent consultants who provide compassionate and quality care to patients for various diseases and conditions. The antenatal clinics at AKUH are run by trained obstetricians specialised in caring for normal and complicated pregnancies. An obstetrician sees 25 to 30 expectant women daily for consultations.

**Fig. 1:**
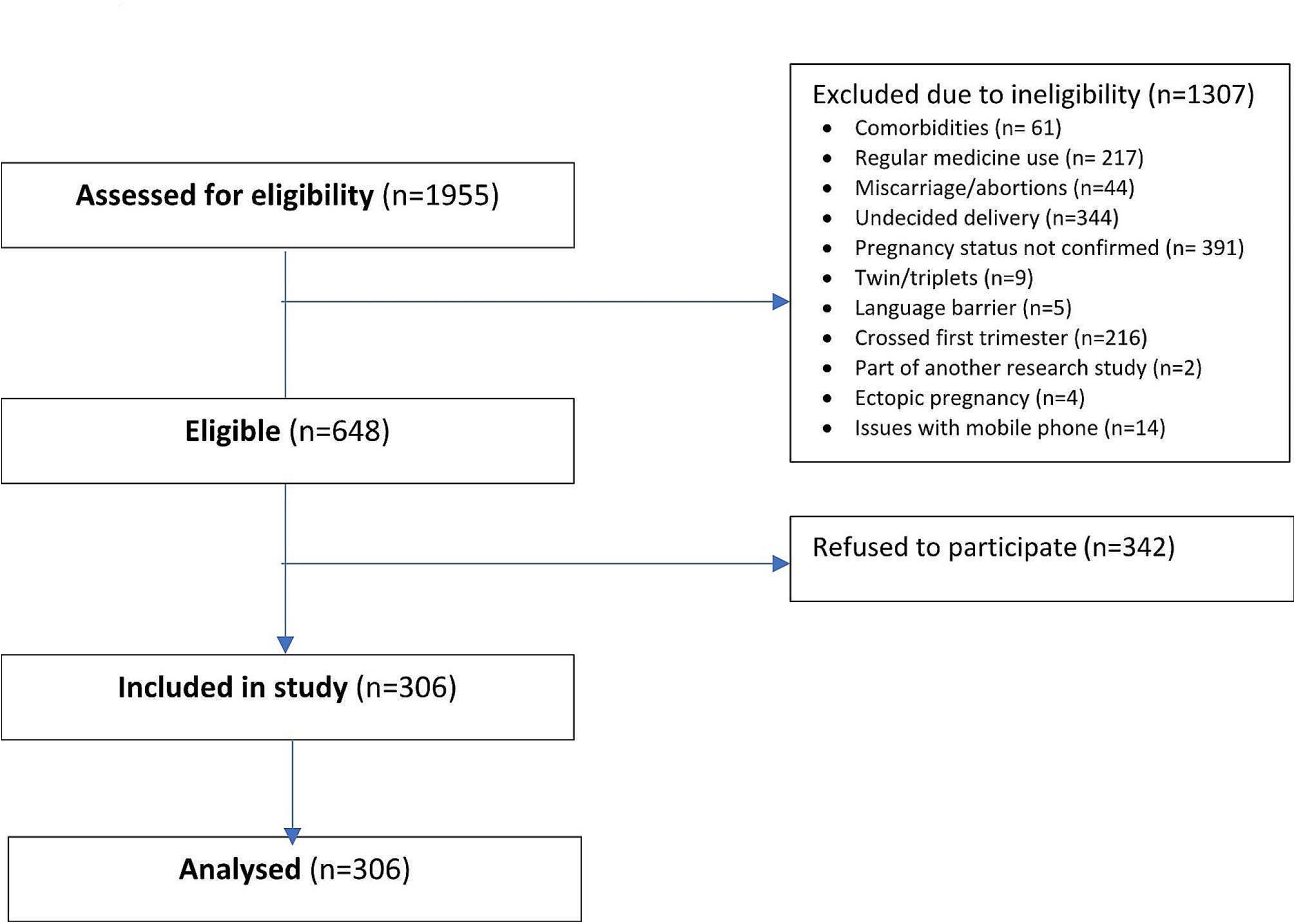
Study flow chart

All pregnant women visiting the antenatal clinics at AKUH during the study duration were eligible for inclusion if they were at least 18 years old, in their first trimester, possessed a personal smartphone with an internet connection (for delivery of a mobile health intervention in the later stage of the study), and agreed to participate. Pregnant women who had comorbidities such as cardiovascular diseases, hypertension, diabetes, autoimmune disorders, kidney or liver diseases, were using medications regularly (antiplatelet aggregators, hypoglycaemic or antihypertensive drugs), and were unable to read and write due to language barriers were excluded. We initially approached 1955 pregnant women for the study; however, 1307 were excluded for various reasons as indicated in Fig. 1. Out of 648 eligible women, 342 did not consent to participate. Ultimately, 306 women were enrolled in the study (Fig. 1).

### Sample size determination and sampling strategy

We calculated a sample size of 306 pregnant women using OpenEpi (version 3.01). The calculation was based on a minimum dietary diversity of 27.6% among WRA [14], an α error of 0.05, and a β error of 0.2, using the formula n = Z^2^ P (1-P)/d^2^, where ‘n’ is the sample size, ‘Z’ is the statistic corresponding to the level of confidence, ‘P’ is prevalence, and ‘d’ is precision [20].

Study participants were identified from the assessment rooms of the antenatal clinics using a purposive sampling strategy to ensure the recruitment of women meeting the eligibility criteria. The clinic list was reviewed a day before to identify the first-trimester pregnant women scheduled for the next day. Potential participants were approached for their written informed consent, and the study objectives were explained in detail to allow an informed decision. Women who agreed to participate were provided with a copy of informed consent.

### Data collection tools and procedures

Through a comprehensive and structured questionnaire (supplementary file 1), we collected data about socio-demographic, obstetric, and dietary characteristics through face-to-face interviews. The socio-demographic information included age, ethnicity, religion, education, and occupation of women and spouses, monthly household income, family structure, house ownership, and household size. The obstetrics information comprised gravida, the experience of nausea, vomiting, and antiemetic use.

For dietary history, we developed a comprehensive food frequency questionnaire with relevance to the Pakistani context. We expanded the questionnaire used in the Smarter Pregnancy Program for Dutch pregnant women and couples contemplating pregnancy [21, 22] by incorporating additional questions about consuming locally available food items within appropriate food groups. Some examples of local food items include paratha (fried flatbread), dahi (yogurt), lassi (buttermilk), laal chawal (red rice), baajre ka aata (pearl millet flour), kaleji (liver), and makai (corn). We collected data about portions (< 1 to ≥ 5) and frequency of consumption over the past week (1 to 7 days) for the various food items, which were classified into the following food groups.

1. Starch-based food (bread, chapatti, bun, naan, paratha, rice, pasta, cereal, corn, and potato).
2. Animal and plant protein (fish, seafood, poultry, red and organ meat, liver, eggs, pulses, legumes, and beans).
3. Vegetable (cooked, raw, fresh, and frozen).
4. Fruit (fresh, dried, frozen, shakes, and fresh juices).
5. Milk and milk products (milk, lassi, yogurt, milkshakes, custard, kheer).
6. Oil and fats (cooking oil, ghee, butter, margarine).

For the oil and fats group, we calculated the monthly cooking oil consumption based on women’s average daily caloric requirement of 2300 calories during pregnancy [23]. Adequate quantity was determined using the guidelines that fats should constitute 30% of the total caloric intake [24], equivalent to 690 calories. Since each gram of fat contains 9 calories [25], 690 calories correspond to approximately 76.66 g of fat or approximately 80 ml. To account for fats from other sources, such as snacks or ready-made foods, we set a limit of 75 ml per day.

We also collected data on snacks, ready-made and homemade meals, beverages, additional salt use, water intake, and food allergies and avoidance. The questionnaire was pretested on 10% of the sample and modified as necessary. We obtained information about booking height, weight, blood pressure, and haemoglobin measurements from the medical records.

The study was implemented by a data collector with extensive experience in antenatal research, along with a doctoral student specializing in maternal nutrition and public health. The data collector was hired and extensively trained over five days to implement the questionnaire, followed by demonstrations, post-training evaluation, and quarterly refresher training. The face-to-face interviews were conducted by the data collector and a doctoral student in a quiet setting such as a treatment room or a secluded area within the clinic’s waiting room, to maintain privacy and confidentiality. Women were guided through each section of the questionnaire and provided assistance as needed. The dietary assessment inquired about the frequency of consumption of items within each food group over the past 7 days. If the response was affirmative, portion sizes ranging from < 1 to ≥ 5 portions per day were determined and recorded accordingly. This process was repeated for each food item across all food groups and for snacks and beverages as well. Women were given ample time to recall information accurately. To minimize recall limitation, a comprehensive list of potential food choices was provided for each food group, supplemented by open-ended questions for additional details. Furthermore, due to the subjective nature of dietary assessment, women were informed of the importance of reporting their actual consumption rather than what they believed should be consumed. Additionally, they were assured that all information provided would be kept confidential and treated without judgement. Model utensils, including plates, bowls, and cups, were utilized to illustrate portion sizes. On an average, the interviews lasted 25–30 min. The data collection initially planned for 6–8 months, was significantly delayed due to the Covid-19 pandemic causing a sharp decline in recruitment, which was stabilized after January 2021.

### Data analysis

The data were entered and analysed using STATA version 14.0. Routine checks for completeness, clarity, and missing information were performed on-site. Based on the normality assessment, for quantitative variables such as age and blood pressure, we report the mean (SD) or medians and IQRs as appropriate. Monthly household income in PKR was categorised as less than 50,000, 50,000–99,999, 100,000–199,999 and 200,000 and above, and household size as ≤ 5 and > 5, respectively. For categorical variables such as ethnicity, religion, house ownership and family income, size and structure, frequency, and percentages are reported.

We assessed dietary quantity and quality using the dietary risk scores (DRS), which were calculated based on the World Health Organization’s (WHO) recommendations for healthy eating during pregnancy and lactation [26]. DRS is a composite score widely used to assess dietary inadequacies based on questions assessing portions and frequency of weekly consumption of items from the six major food groups. In our study, we extended the DRS in addition to fruit and vegetable intake to other main food groups (Table 1), following a similar approach as van Dijk et al., 2020. For each food group, women can score 0 (adequate), 1.5 (intermediate), or 3 (inadequate) separately for dietary quantity and quality [27]. The total score based on the sum of individual food group scores could range from 0 to 18, where 18 indicates an inadequate diet for all food groups.

**Table 1:** Dietary risk score (DRS) for six food groups

Food groups	Dietary Risk Score		
	0	1.5	3
**Starch-based Food**			
Daily quantity	≥ 6.0 portions	3.0 to 5.9 portions	< 3.0 portions
Quality	≥ 50% of whole grains	25 to 49% of whole grains	< 25% of whole grains
**Fruit**			
Daily quantity	≥ 2.0 portions	1.0 portion	< 1.0 portion
Quality	Whole fruit consumption	< 50% of whole fruits and > 50% as shakes or fruit juices	Juices and/or shakes only
**Vegetable**			
Daily quantity	≥ 3.0 portions	1.5 to 2.9 portions	< 1.5 portions
Quality	> 1/3 raw and < 2/3 cooked	< 1/3 raw and > 2/3 cooked	Only in one form
**Animal and Plant Protein**			
Daily quantity	2.0 portions	1.0 portion	< 1.0 portion
Quality	All sources (fish and meat 2 times/week; plant based protein ≥ 4 times/week)	Fish > 2 or < 1 times/week; meat > 2 or < 1 times/week; plant-based protein 2 to < 4 times/week	No fish; no plant-based protein; only meat
**Milk and Milk Products**			
Daily quantity	3.0 portions	1.5 to 2.9 portions	< 1.5 portions
Quality	Milk and dairy products	Milk or dairy products	None
**Oils and Fats**			
Daily quantity	≤ 30% of total calories	31–35% of total calories	> 35% of total calories
Quality	Polyunsaturated	Monounsaturated	Saturated

### Ethical considerations

The study was approved by the Ethics Review Committee of Aga Khan University (Reference Number 2021-5749-18595). Written informed consent was obtained from all participating women.

## Results

### Socio-demographic characteristics

The study included 306 pregnant women with a mean age of 28.4 years (range of 18–45 years). Most were Muslim (96.4%) and belonged to the Mohajir ethnicity (48%). A majority had attained university-level education, but only a third were employed. Most women lived in owned houses (72.9%) and in extended family arrangements (80.1%). Two-thirds of the sample reported their monthly household income, with over half earning less than 100,000 PKR. The minimum wage in Pakistan from 2019 to 2021 was 17,500 PKR per month [28]. Spouses generally had higher educational and employment statuses than women (Table 2).

**Table 2:** Socio-demographic characteristics (*N* = 306)

Characteristics	Mean ± SD *n* (%)
**Age** (years)	28.4 ± 4.2
**Age**	
< 30 years ≥ 30 years	191 (62.4) 115 (37.6)
**Ethnicity**	
Sindhi Punjabi Pakhtoon Mohajir Memon/Gujarati Others*^1^	57 (18.6) 42 (13.7) 11 (3.6) 147 (48.0) 32 (10.5) 17 (5.5)
**Religion**	
Muslim Non-Muslim	295 (96.4) 11 (3.6)
**Educational status**	
Up to intermediate University and above	67 (21.9) 239 (78.1)
**Educational status of spouse**	
Up to intermediate University and above	25 (8.2) 281 (91.8)
**Employment status**	
Employed Unemployed	104 (34) 202 (66)
**Occupation** (*n* = 104)	
Commerce (Administration/Human Resource/Finance) Health care professionals Teacher/Faculty Engineer/Architect Allied Health Professionals Self-employed Other*^2^	30 (28.8) 29 (27.9) 13 (12.5) 11 (10.6) 7 (6.7) 8 (7.7) 6 (5.8)
**Employment status of spouse**	
Employed	306 (100)
**Household income PKR/month** (*n* = 207)	
Less than 50,000 50,000–99,999 100,000–199,999 200,000 and above	29 (14.0) 104 (50.2) 51 (24.6) 23 (11.1)
**Status of housing**	
Owned Rented	223 (72.9) 83 (27.1)
**Family structure**	
Nuclear Extended	61 (19.9) 245 (80.1)
**Household members**	
≤ 5 > 5	156 (51) 150 (49)

*^1^ Balochi (*n* = 5); Bohri (*n* = 4); Gilgity (*n* = 4); Gojali (*n* = 1); Kachi (*n* = 2); Saraiki (*n* = 1)

*^2^ Social activist (*n* = 1); Researcher (*n* = 2); not reported (*n* = 3)

SD: standard deviation, PKR: Pakistani rupees

### Obstetric history and physical assessment

The gestational age of women ranged from 4.4 to 13.4 weeks. Our sample had slightly more multigravida women. Over half experienced nausea and vomiting, but only a third used antiemetics. Additionally, a greater proportion were obese. Blood pressure and haemoglobin levels were generally normal, however, one-fourth were anaemic (Table 3).

**Table 3:** Obstetric history and physical assessment (*N* = 306)

Characteristics	*n* (%)
**Gravida**	
Multigravida Primigravida	172 (56.2) 134 (43.8)
**Nausea**	213 (69.6)
**Vomiting**	168 (54.9)
**Use of antiemetic**	109 (35.6)
**BMI** (Kg/m^2^)*	
Underweight (< 18.5) Normal (18.5–22.9) Overweight (23-24.9) Obese (≥ 25)	24 (7.8) 90 (29.4) 59 (19.3) 133 (43.5)
**Blood pressure** (mmHg) mean ± SD	
Systolic Diastolic	115.7 ± 11.8 70.4 ± 8.2
**Haemoglobin** (gm/dl) median (IQR) (*n* = 173)	11.7 (11, 12.4)
**Anaemia** (Hb < 11 gm/dl) (*n* = 173)	42 (24.3)

SD: standard deviation, IQR: interquartile range, BMI: body mass index

*WHO Asian-BMI classification [29]

### Dietary intake of pregnant women during the first trimester

**Fig. 2:**
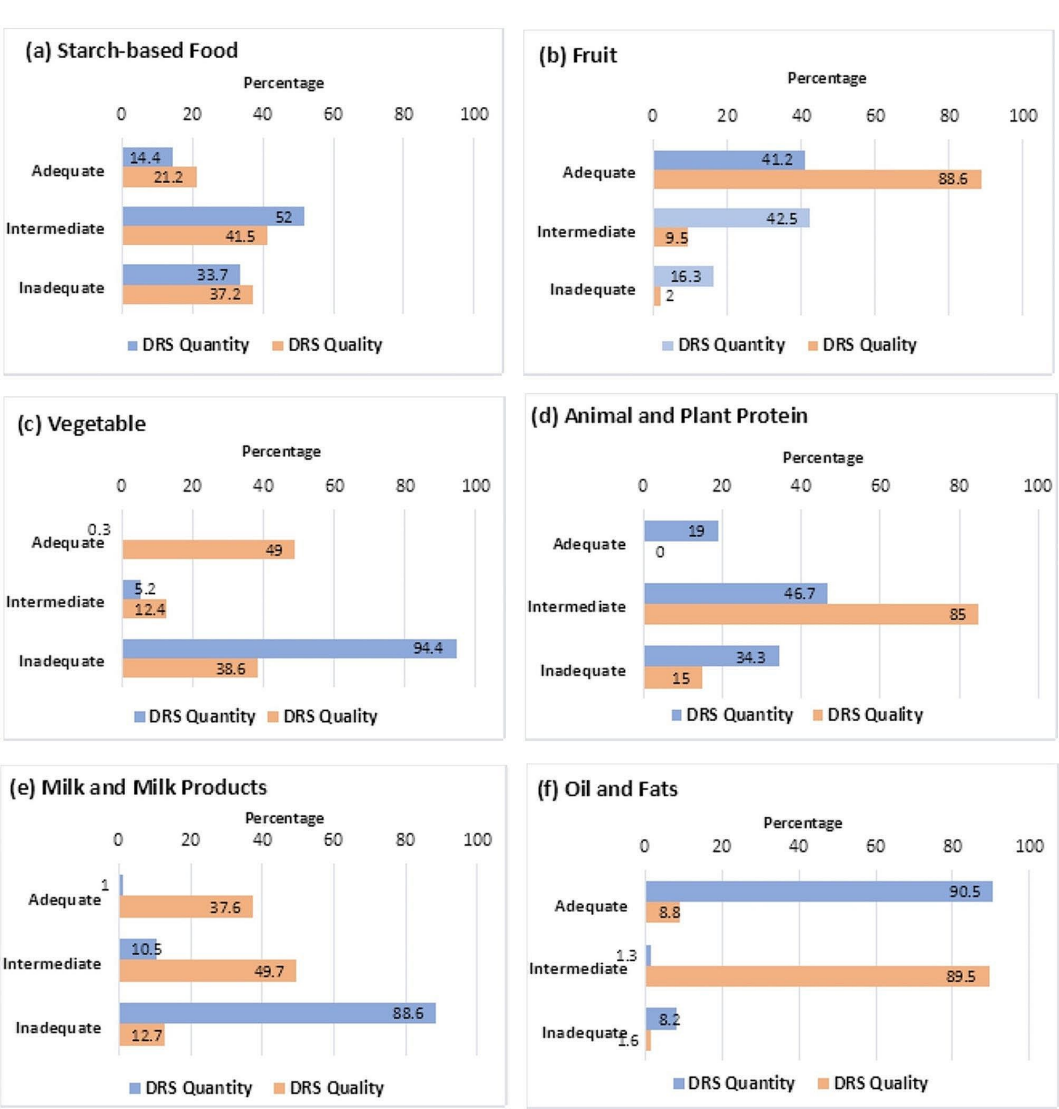
Dietary risk scores for the quantity and quality of six food groups. Proportion of women scoring adequate, intermediate and inadequate for the quantity and quality of (**a**) starch-based food (**b**) fruit (**c**) vegetable (**d**) animal and plant protein (**e**) milk and milk products (**f**) oil and fats

The mean ± SD DRS for quantity and quality were 10.6 ± 2.4 and 7.5 ± 2.5, respectively. Oil and fats (90.5%) had the highest adequate intake, while vegetables and milk products were the lowest at 0.3% and 1%, respectively. Regarding dietary quality, 88.6% of the participants scored adequately for fruit, but none for animal and plant proteins (Fig. 2). Around 5.6% reported food allergies to meat, eggs, milk/cheese, nuts/peanuts, fruits, honey, and mushrooms, while 3.9% avoided meat, vegetables, fish/seafood, and milk in their diet. Most women ate three homemade meals daily, drank a median of six glasses of water, and a few used additional salt in their food.

### Dietary intake of pregnant women for the six food groups

#### Starch-based food

White rice was the most consumed item, followed by whole wheat chapatti, potato, white bread/bun, and paratha. Most of these items were consumed once daily, except for paratha, which was usually eaten in at least four portions. Whole grain intake, apart from whole wheat chapatti, was very low (Table 4).

**Table 4:** Consumption of items from five main food groups (*N* = 306)

Food groups	*n* (%)^a^	Frequency (Days/week)			Quantity (Portions/day)		
		1–2	3–5	6–7	≤ 1	2–3	≥ 4
		*n* (%)^b^	*n* (%)^b^	*n* (%)^b^	*n* (%)^b^	*n* (%)^b^	*n* (%)^b^
**Starch-based Food**							
White bread/bun/double roti	226 (73.9)	78 (34.5)	60 (26.5)	88 (38.9)	177 (78.3)	48 (21.2)	1 (0.4)
Brown bread/whole wheat bread	43 (14.0)	15 (34.9)	13 (30.2)	15 (34.9)	31 (72.1)	12 (27.9)	0 (0)
Whole wheat chapatti	266 (86.9)	21 (7.9)	48 (18.0)	197 (74.1)	186 (69.9)	77 (28.9)	3 (1.1)
White flour chapatti/naan	139 (45.4)	100 (71.9)	25 (18.0)	14 (10.1)	75 (54.0)	59 (42.4)	5 (3.6)
Paratha	185 (60.5)	102 (55.1)	33 (17.8)	50 (27.0)	15 (8.1)	27 (14.6)	143 (77.3)
White rice	300 (98.0)	99 (33.0)	124 (41.3)	77 (25.7)	270 (90.0)	24 (8.0)	6 (2)
Brown rice	9 (2.9)	8 (88.9)	1 (11.1)	0 (0)	9 (100)	0 (0)	0 (0)
Noodles/pasta	141 (46.1)	129 (91.5)	11 (7.8)	1 (0.7)	130 (92.2)	8 (5.7)	2 (1.4)
Cereal	46 (15.0)	27 (58.7)	14 (30.4)	5 (10.9)	46 (100)	0 (0)	0 (0)
Potato	255 (83.3)	124 (48.6)	94 (36.9)	37 (14.5)	232 (91.0)	23 (9.0)	0 (0)
Corn	50 (16.3)	36 (72.0)	9 (18.0)	5 (10.0)	49 (98.0)	1 (2.0)	0 (0)
**Fruit**							
Raw fruits	288 (94.1)	20 (6.9)	52 (18.0)	216 (75.0)	249 (86.5)	36 (12.5)	3 (1.0)
Other fruits	89 (29.1)	43 (48.3)	18 (20.2)	28 (31.5)	83 (93.2)	6 (6.7)	0 (0)
Tinned fruits	9 (2.9)	6 (66.7)	1 (11.1)	2 (22.2)	9 (100)	0 (0)	0 (0)
Nuts/dry fruits	160 (52.3)	42 (26.2)	29 (18.1)	89 (55.6)	152 (95.0)	8 (5.0)	0 (0)
Fruit shakes	101 (33.0)	57 (56.4)	25 (24.7)	19 (18.8)	100 (99.0)	1 (1.0)	0 (0)
Fresh fruit juices	138 (45.1)	66 (47.8)	37 (26.8)	35 (25.4)	130 (94.2)	7 (5.1)	0 (0)
**Vegetable**							
Cooked vegetables	254 (83.0)	96 (37.8)	124 (48.8)	34 (13.4)	249 (98.0)	5 (2.0)	0 (0)
Raw vegetables	214 (69.9)	64 (29.9)	58 (27.1)	92 (43.0)	213 (99.5)	1 (0.5)	0 (0)
**Animal and Plant Protein**							
Fish	75 (24.5)	71 (94.7)	4 (5.3)	0 (0)	70 (93.3)	5 (6.7)	0 (0)
Red meat	220 (71.9)	131 (59.5)	70 (31.8)	19 (8.6)	207 (94.1)	12 (5.4)	1 (0.4)
Poultry	233 (76.1)	124 (53.2)	86 (36.9)	23 (9.9)	202 (86.7)	30 (12.9)	1 (0.4)
Liver	9 (2.9)	8 (88.9)	1 (11.1)	0 (0)	9 (100.0)	0 (0)	0 (0)
Organ meat	2 (0.6)	2 (100.0)	0 (0)	0 (0)	2 (100.0)	0 (0)	0 (0)
Seafood	12 (3.9)	12 (100.0)	0 (0)	0 (0)	11 (91.7)	0	1 (8.3)
Egg	221 (72.2)	61 (27.6)	63 (28.5)	97 (43.9)	216 (97.7)	5 (2.3)	0 (0)
Plant-based sources	248 (81.0)	179 (72.2)	53 (21.4)	16 (6.4)	236 (95.2)	12 (4.8)	0 (0)
**Milk and Milk Products**							
Milk	197 (64.4)	29 (14.7)	39 (19.8)	129 (65.5)	184 (93.4)	13 (6.6)	0 (0)
Milk products	183 (59.8)	67 (36.6)	67 (36.6)	49 (26.8)	180 (98.4)	3 (1.6)	0 (0)

^a^ % calculated in column 2 are column %

^b^ % calculated in column 3–8 are row %

#### Fruit

Most women consumed fruits daily and half reported eating nuts and dried fruits regularly. Nearly half of the sample consumed fresh fruit juices and one-third had milkshakes up to twice a week, usually in a single portion (Table 4).

#### Vegetable

The habit of eating cooked vegetables was more common than that of raw vegetables. Most women ate no more than one portion daily (Table 4).

#### Animal and Plant Protein

Red meat, poultry, and eggs were the most consumed items from animal sources. Eggs were eaten daily by a higher proportion of women, while only a quarter consumed fish no more than twice a week. Plant-based sources such as pulses, legumes, and beans were consumed by most women, but not more than twice a week. Other food items, such as seafood, liver, and organ meat were less commonly eaten (Table 4).

#### Milk and milk product

Most women consumed milk more frequently than milk products, typically limiting their intake to one portion per day (Table 4).

#### Oil and Fats

Nearly all women used cooking oil, while a few used clarified butter (ghee). Canola was most common, followed by soybean, and olive oil. Mono-saturated fat consumption predominated.

### Consumption of unhealthy food items: snacks, ready-made meals, and beverages

Over half of the sample consumed ready-made meals and snacks, with sweet snacks more common than savoury ones. Tea was the most consumed beverage, followed by carbonated drinks. One in three women reported consuming packaged juices (Table 5).

**Table 5:** Consumption of snacks, ready-made meals, and beverages by pregnant women (*N* = 306)

Food items	*n* (%)^a^	Frequency (Days/week)			Quantity (Portions/day)		
		≤ 2	3–5	6–7	≤ 1	2–3	≥ 4
		*n* (%)^b^	*n* (%)^b^	*n* (%)^b^	*n* (%)^b^	*n* (%)^b^	*n* (%)^b^
Savoury snacks	165 (53.9)	131 (79.4)	21 (12.7)	13 (7.9)	131 (79.4)	31 (18.8)	3 (1.8)
Sweet snacks	229 (74.8)	157 (68.5)	48 (20.9)	24 (10.5)	210 (91.7)	18 (7.9)	1 (0.4)
Ready-made meals	169 (55.2)	147 (87.0)	18 (10.6)	4 (2.4)	149 (88.2)	17 (10.1)	3 (1.8)
Packaged juice	105 (34.3)	63 (60.0)	26 (24.8)	16 (15.2)	95 (90.5)	9 (8.6)	1 (0.9)
Tea	188 (61.4)	10 (5.3)	8 (4.2)	170 (90.4)	130 (69.1)	55 (29.2)	3 (1.6)
Coffee	20 (6.5)	17 (85.0)	2 (10.0)	1 (5.0)	20 (100.0)	0 (0)	0 (0)
Carbonated beverages	143 (46.4)	88 (61.5)	39 (27.3)	16 (11.2)	140 (97.9)	3 (2.1)	0 (0)

^a^ % calculated in column 2 are column %

^b^ % calculated in column 3–8 are row %

## Discussion

This cross-sectional study provides a comprehensive snapshot of the dietary habits of first-trimester pregnant women attending a tertiary care hospital in Karachi, Pakistan. Our findings indicate that, on average, these women do not adhere to recommended dietary guidelines, leading to inadequate and imbalanced diets. Starch-based food, vegetables, animal and plant protein, and milk and milk products were insufficiently consumed, and dietary quality was inadequate for all food groups except fruits.

Starch-based food plays a crucial role in supporting maternal health and fetal development [26]; however, we found that only 14.4% of women met the recommended intake (≥ 6 portions). This finding is consistent with low compliance reported in studies from Thailand (13.2%) [30], Canada (8.6%) [31], and New Zealand (26%) [32]. In contrast, 98% of Indian pregnant women predominantly consumed starch-based foods [33]. Quality-wise, only 21% of women achieved an adequate score, including items like corn (16.3%), brown bread (14%), and brown rice (2.9%). We also observed excessive use of refined sources, in particular white rice (98%), white bread/bun (73.9%), paratha (60.5%), and noodles/pasta (46.1%), comparable to the study findings from Lahore [34].

Although whole grain consumption was similarly low in Singapore (29.6%) [35] and Iceland (14.9%) [36], recent research in rural Sindh found two-thirds of pregnant women mainly consumed brown rice or wheat-flour chapattis [37]. In Gujarat, India, commonly consumed sources included rice, sorghum, millet, wheat, and semolina [33], suggesting better choices, though processing details were not provided. The low whole grain intake may result from limited awareness of their health benefits [38], perceived higher cost, taste preferences, or family choices [39]. Nutrition counselling should promote adequate consumption and raise awareness about superior health benefits of whole grains in reducing pregnancy complications, obesity, excessive gestational weight gain (GWG), and constipation [1].

Being rich in essential vitamins, minerals, and dietary fibre, daily fruit consumption reduces the risk of adverse pregnancy outcomes [40]. While most women in our study (98.7%) consumed fruits regularly, only 41.2% met the recommended quantity (≥ 2 portions). Similar patterns were found in China (77.5%) [41], India (59%) [33], and the Netherlands (56.9% with DRS 0) [27]. In contrast, pregnant women in Southern India exceeded recommended fruit intake by threefold [42], while lower consumptions were observed in Nepal [43], Spain [44], Australia [45], and Canada [7]. Compared to pregnant women in rural Sindh (< 3%) [37] and Lahore (59%) [34], our sample had higher fruit intake and quality but also consumed more fruit juices (44.8% vs. 23%) [34]. Thus, nutrition counselling should encourage seasonal fruit and nut consumption while discouraging excessive fruit juice intake.

Vegetables are an essential part of a healthy diet to support normal physiological functions and prevent adverse outcomes during pregnancy [46]. We observed that 91.5% of women consumed vegetables weekly, higher than reported among pregnant women in Lahore (54%) [34]. However, only 0.3% met the recommended quantity (≥ 3 portions/day), and 49% met the recommended quality. These findings align with studies from India [47], Nepal [43], Bangladesh [48, 49], and rural Malawi [50]. In comparison, the Western population reported more vegetable consumption [27, 31]. In Pakistan, as observed in rural Sindh (1%) [37] and in Rawalpindi (2%) [51], vegetable consumption is extremely low. The inadequate vegetable intake may result from factors such as taste preferences, traditional beliefs favouring calorie-dense food, family customs prioritising other foods and limited awareness of the health benefits. Addressing these factors during antenatal care is important to promote recommended vegetable intake.

Similarly, protein is a major macronutrient required to support blood volume expansion, growth of maternal and fetal tissues, and placental development [52]. However, in our study, both the quantity (18.5%) and, notably, the quality (0%) of protein intake fell below the recommended levels. Similar findings are reported by Morton et al., where only 21% of women met the recommendations [32]. In contrast, higher than recommended protein intake was reported among first-trimester pregnant women in China [41, 53]. Regarding different protein sources, our study showed similar consumption of lentils/beans (81% vs. 84%) compared to Lahori pregnant women [34] but greater meat (90% vs. 75%), eggs (72% vs. 60% weekly; 30% vs. 4% daily) and fish (25% vs. 0%) consumption [34]. We also found a higher poultry intake (76.1%) compared to pregnant women in Rawalpindi (14%) [51]. Despite these improved dietary practices, women in our study did not meet adequate dietary quality requirements.

Furthermore, milk and dairy products are nutrient-rich sources, and their adequate intake during pregnancy improves fetal growth, development, and birth weight and size [54]. While over half of our sample consumed them weekly, only 1% met the recommended quantity, and 37.6% met the recommended quality. Our findings are consistent with previous studies from various countries, including China [41], Iran [55], India [56], Spain [57], LMICs [37], and Pakistan [34]. Conversely, in Gujarat, India, a higher proportion of pregnant women (82.6%) reported dairy consumption, but adequacy was not established [33]. A similar pattern of higher dairy intake during pregnancy compared to non-pregnant women was reported in Belgium [58] and New Zealand [32]. Insufficient dairy consumption may arise due to typical dietary patterns and poor recognition of its significance during pregnancy, hence requiring nutrition counselling to encourage adherence to recommended consumption.

Dietary fats, particularly polyunsaturated fatty acids (PUFAs), are important in supporting energy needs and promoting fetal development [59, 60]. Since the human body cannot synthesise PUFAs, their inclusion in the diet through sources such as fish is essential. Nutritionists also stress the significance of vegetable oils due to their rich content of beneficial fatty acids [61]. In our study, most women (90.52%) met the recommended quantity for oil and fats, but only 8.8% met the recommended quality. Monounsaturated fatty acids (MUFAs) were more commonly consumed (89.5%) than PUFAs, reporting similar patterns in India [62], Spain [57], and Belgium [58]. However, a Thai study found that 78% of pregnant women had inadequate fat intake [30].

In addition to major food groups, our study also evaluated the consumption of unhealthy dietary items. In this regard, we found consumption patterns similar to the Western world [32, 63], with comparable consumption of sweet (74.8% weekly; 10% daily) and savoury snacks (53.9% weekly; 8% daily) and carbonated beverages (Weekly 50%; 11.3% daily). However, we observed a higher intake of ready-made meals (55.2% weekly; 4% daily). Contrastingly, a Singaporean study involving different ethnicities showed reduced consumption of confectioneries during pregnancy [64].

In our study, women reported a median daily water intake of 6 glasses, comparable to Australian pregnant women [65] and higher than Chinese pregnant women [66]. Tea was more frequently consumed (61.4%) than coffee (6.5%) on most days, contrasting with Chinese data showing low tea consumption in the first trimester [67]. It is important to note that tea can reduce iron absorption, and a quarter of our participants were anaemic, emphasising the need for quality nutrition counselling to minimise absorption interference. Furthermore, only 3.9% of women reported food avoidance for religious and cultural reasons, much lower than reported in China (80%) [68], Tanzania (70.1%) [69], and other settings [58].

### Strengths and limitations

This is one of the few studies in Pakistan to evaluate the dietary adequacy of pregnant women during the first trimester. We individually assessed both the quantity and quality of six major food groups, considering locally available and seasonal options. We developed a DRS using a comprehensive dietary algorithm, aligning with dietary guidelines, and gathered detailed insights into participants’ consumption patterns through open-ended questions.

However, the study has several limitations that need to be acknowledged. First, the cross-sectional study design limits tracking dietary inadequacies during preconception and provides a snapshot of dietary status at one point in time. Another limitation is related to the generalizability of the findings. Our sample was selective, comprising women with personal smartphones (for the planned RCT) who attended antenatal clinics at a private tertiary care hospital indicating a relatively better socio-economic standing than the general population, which might influence their dietary habits and access to healthcare resources. Despite the selective sample, we identified dietary inadequacies across major food groups, potentially suggesting significant deficiencies in pregnant women from impoverished backgrounds. Furthermore, we observed that a greater proportion of eligible women opted out of participating in the study, potentially leading to non-response bias, suggesting that their characteristics may differ from those of the respondents. Therefore, the results may not be representative of the broader population and should be interpreted within the context.

In addition, we anticipated recall bias concerning dietary information collected over the past one week. However, we minimized it by obtaining the data using an item checklist and included open-ended questions to capture any additional information. Further, due to cost and other constraints, we could not verify the dietary assessment through biochemical nutrient evaluation; although it is not possible to biochemically assess all nutrients. Lastly, seasonal variations could have impacted dietary quantity and diversity across the study participants, which we have not taken into account.

### Implications

The findings of our study have several important implications. First, they reveal dietary inadequacies during the first trimester, indicating a need for targeted public health interventions early in pregnancy. Additionally, these findings relate to women from better socioeconomic backgrounds, implying the existence of potentially greater deficiencies among those from impoverished backgrounds. Hence, there is a need for widespread awareness campaigns and quality nutritional counselling as early as the first trimester. Secondly, there is a pressing need for nutritional guidelines specific to the Pakistani population, incorporating local food choices and tailored to context-specific dietary requirements for different stages of life including pregnancy. Further, innovative methods for monitoring dietary habits, providing repeated feedback and ensuring compliance with the recommendations should be explored and tested for effectiveness to ensure personalized and effective nutritional support during pregnancy.

## Conclusion

Overall, first-trimester pregnant women consume an inadequate diet. Substantial inadequacies are evident in the amount and quality of starch-based food, animal and plant protein, and milk and milk products. Vegetable intake falls below the recommendations, while the quality of oils and fats is suboptimal. Addressing maternal malnutrition is critical to achieving SDG and promoting offspring’s growth and development during gestation and beyond. Hence, antenatal nutrition counselling in the first trimester should emphasise adequate and diverse choices from various food groups, which should be maintained throughout the subsequent trimesters. Additionally, potentially innovative and impactful approaches such as employing digital technology in creating awareness and promoting healthy dietary choices can be explored for personalised dietary counselling during the antenatal period.

### Electronic supplementary material

Below is the link to the electronic supplementary material.

Supplementary Material 1

## Data Availability

The datasets used and/or analysed during the current study are available from the corresponding author upon reasonable request.

## Abbreviations

AKUH: Aga Khan University Hospital
BMI: Body Mass Index
CI: Confidence Interval
DRS: Dietary Risk Score
GDM: Gestational Diabetes Mellitus
IQR: Interquartile Range
LBW: Low birth weight
LMICs: Low-and Middle-Income Countries
MUFAs: Monounsaturated fatty acids
PKR: Pakistani Rupees
PUFAs: Polyunsaturated fatty acids
SD: Standard deviation
SDGs: Sustainable Development Goals
WHO: World Health Organization
WRA: Women of reproductive age
