# Supplementary material for: Are first-trimester pregnant women consuming adequate and diverse diet? A hospital-based cross-sectional study in Karachi, Pakistan
Source: BMC Nutr. 2024 Jul 24;10:104. doi: 10.1186/s40795-024-00912-3 (PMC11270889; doi:10.1186/s40795-024-00912-3)
Supplement: Supplementary file 1 — Supplementary Material 1 [file 40795_2024_912_MOESM1_ESM.doc]

**Study Questionnaire**

| **Section A: Eligibility and Consent** | | | |
| --- | --- | --- | --- |
| A1 | Are you pregnant? | **⬜ Yes** | **⬜ No** |
| A2 | What was the first day of your last menstrual period (LMP)  If you are uncertain about the date, provide an approximate date. | **dd/mm/yy _________** | |
| A3a | Expected Date of Delivery according to LMP | **dd/mm/yy _________** | |
| A3b | Expected Date of Delivery according to Ultrasound | **dd/mm/yy _________** | |
| A4 | Gestational Age at the time of enrollment | **Days ____________** | |
| A5 | Do you have a personal smartphone or mobile phone with internet connection? | **⬜ Yes** | **⬜ No** |
| A6 | Do you agree to participate in this research project according to the local regulations? | **⬜ Yes** | **⬜ No** |
| A7 | Can you read and write in English or Urdu? | **⬜ Yes** | **⬜ No** |
| ***If the answer to any of above questions is “No,” the woman is not eligible for study.*** | | | |
| A8 | Do you have diabetes? | **⬜ Yes** | **⬜ No** |
| A9 | Do you have cardiovascular disease (hypertension, angina)? | **⬜ Yes** | **⬜ No** |
| A10 | Are you on dietary control due to co-morbid such as Diabetes or Cardiovascular Diseases? | **⬜ Yes** | **⬜ No** |
| A11 | Are you on medications such as anti-hypertensive drugs? | **⬜ Yes** | **⬜ No** |
| A12 | Are you on any diabetic medications such as Glucophage? | **⬜ Yes** | **⬜ No** |
| A13 | Are you on medications such as Ascard? | **⬜ Yes** | **⬜ No** |
| A14 | Do you have any serious medical conditions such as kidney or liver diseases, SLE? | **⬜ Yes** | **⬜ No** |
| ***If answer to any of above questions is “Yes,” woman is not eligible for study.*** | | | |
| A15 | Is woman eligible to participate? | **⬜ Yes** | **⬜ No** |
| A16 | Has she read, understood and signed the Informed Consent? | **⬜ Yes** | **⬜ No** |
| ***If answer to all of the above questions is “Yes,” woman can be enrolled as the study subject.*** | | | |

| **Section B: Participant information** | | | | | | | | | | | | |
| --- | --- | --- | --- | --- | --- | --- | --- | --- | --- | --- | --- | --- |
| **S.no** | **Questions** | **Responses** | | | | | | | | | | |
| B1 | Date (dd/mm/yy) |  | | | | | | | | | | |
| B2 | Study ID # |  | | | | | | | | | | |
| B3 | Medical Record (MR) Number |  | | | | | | | | | | |
| B4 | Name |  | | | | | | | | | | |
| B5 | Husband/Father’s name: |  | | | | | | | | | | |
| B6 | Ethnicity | 1. Sindhi | 2. Punjabi | | | 3. Balochi | | 4. Pakhtoon | | | | 5. Mohajir |
|  |  | 6. Memon | 7. Gujarati | | | 8. Others (Specify): ______________ | | | | | | |
| B7 | Religion | 1. Islam 2. Hinduism 3. Christianity 4. Other (specify): ______________ | | | | | | | | | | |
| B8 | Address |  | | | | | | | | | | |
| B9 | Zip/Area code |  | | | | | | | | | | |
| B10 | Contact number (home and cell) | Home: _______________  Cell number: _________________ | | | | | | | | | | |
| B11 | Date of Birth (dd/mm/yy) |  | | | | | | | | | | |
| B12 | Age (in years) |  | | | | | | | | | | |
| B13 | Your level of education | 1. Uneducated | | 2. Primary | | | | | 3. Secondary | | | |
|  |  | 4. College | | 5. University | | | | |  | | | |
| B14 | Husband’s level of education | 1. Uneducated | | 2. Primary | | | | | | 3. Secondary | | |
|  |  | 4. College | | 5. University | | | | | | 6. Not Applicable | | |
| B15. | Your Occupation  (*Skip Question B15a, if response is unemployed)* | 1. Employed | | 2. Unemployed | | | | | | 3. Self-employed | | |
| B15a | If employed, what is your profession? |  | | | | | | | | | | |
| B16 | Husband’s occupation | 1. Employed  4. Not Applicable | | | 2. Unemployed | | | | | | 3. Self-employed | |
| B17 | Monthly family income | 1. <50,000 2. 50,000 – <100,000 3. 100,000 – 200,000 4. 200,000 – 500,000 5. > 500,000 6. Not answered | | | | | | | | | | |
| B18 | Status of housing | 1. Owned | |  | | | 2. Rented | | | | | |
| B19 | Type of family | 1. Nuclear | | | | | 2. Extended | | | | | |
| B20 | Number of members in the family | _________ Members | | | | | | | | | | |

| **Section C: General Food Information** | | | |
| --- | --- | --- | --- |
| C1 | Do you have any food allergies?  *(Skip question C1a if response to this question is No or Do Not Know)* | 1. Yes 2. No 3. Do Not Know | |
| C1a | Which food are you allergic to? | 1. Bread, rice, cereal, pasta and potato group | Specify: |
|  |  | 1. Fruits | Specify: |
|  |  | 1. Vegetables | Specify: |
|  |  | 1. Meat and meat products | Specify: |
|  |  | 1. Milk and milk products | Specify: |
|  |  | 1. Oil and fats | Specify: |
| C2 | Do you have any cultural or religious preference for food consumption?  *(Skip question C2a if response to this question is No)* | 1. Yes 2. No | |
| C2a | What food preference do you have? | 1. Bread, rice, cereal, pasta and potato group | Specify: |
|  |  | 1. Fruits | Specify: |
|  |  | 1. Vegetables | Specify: |
|  |  | 1. Meat and meat products | Specify: |
|  |  | 1. Milk and milk products | Specify: |
|  |  | 1. Oil and fats | Specify: |
| C3 | On an average, how many meals a day do you eat home cooked food?  (Meals refer here to breakfast, lunch and dinner. For snacks, specify in other) | 1. One 2. Two 3. Three 4. Other (Specify) ___________ | |

| **Section D: Anthropometric and blood pressure measurement at the first visit: To be obtained from medical records** | | |
| --- | --- | --- |
| **S.no** | **Questions** | **Responses** |
| D1 | Date of Assessment (dd/mm/yy) |  |
| D2 | Height (in cms) |  |
| D3 | Weight (in kg) |  |
| D4 | Blood pressure (mmHg) | Systolic ____________ Diastolic _____________ |

| **Section E: Obstetrics History** | | | | | |
| --- | --- | --- | --- | --- | --- |
| **S.no** | **Questions** | | **Responses** | | |
| E1 | Number of previous pregnancies | |  | | |
| E2 | Number of live births | |  | | |
| E3 | Do you have nausea related to pregnancy? | | 1. Yes | 2. No | |
| E4 | Do you have vomiting related to pregnancy? | | 1. Yes | 2. No | |
| E5 | Are you taking any antiemetic for nausea or/and vomiting? | | 1. Yes | 2. No | |
|  | | | | | |
| | **Section F: Screening for Nutritional Habits** | | --- | | | | | | |
| **Section F Part 1: Bread, rice, cereal, pasta and potato group** | | | | | |
| F1.1 | | Last week, how many DAYS did you eat white bread/ double rotti/ bun?  *(Skip question I1.1a, if response is 0)* | | | 0 1 2 3 4 5 6 7 |
| F1.1a | | How many PORTIONS did you eat per day?  **One portion size means:**  **1 bread slice**  **1 double rotti**  **½ bun (fruit or plain)** | | | <1 1 2 3 4 >=5 |
| F1.2 | | Last week, how many DAYS did you eat brown bread or whole wheat bread?  *(Skip question I1.2a, if response is 0)* | | | 0 1 2 3 4 5 6 7 |
| F1.2a | | How many PORTIONS did you eat per day?  **One portion size means 1 large bread slice** | | | <1 1 2 3 4 >=5 |
| F1.3 | | Last week, how many DAYS did you eat whole wheat flour chapatti/ baajrey Ki roti?  *(Skip question I1.3a, if response is 0)* | | | 0 1 2 3 4 5 6 7 |
| F1.3a | | How many PORTIONS did you eat per day?  **One portion size means:**  **1 chapatti (6 inch)** | | | <1 1 2 3 4 >=5 |
| F1.4 | | Last week, how many DAYS did you eat white flour chapatti or naan?  *(Skip question I1.4a, if response is 0)* | | | 0 1 2 3 4 5 6 7 |
| F1.4a | | How many PORTIONS did you eat per day?  **One portion size means:**  **1 chapatti (6 inch)**  **Naan (half)** | | | <1 1 2 3 4 >=5 |
| F1.5 | | Last week, how many DAYS did you eat paratha?  *(Skip question I1.5a, if response is 0)* | | | 0 1 2 3 4 5 6 7 |
| F1.5a | | How many PORTIONS did you eat per day?  **One portion size means 1 quarter paratha** | | | <1 1 2 3 4 >=5 |
| F1.6 | | Last week, how many DAYS did you eat rice?  *(Skip question I1.6a if response is 0)* | | | 0 1 2 3 4 5 6 7 |
| F1.6a | | How many PORTIONS did you eat per day?  **One portion size means half bowl of rice (cooked)** | | | <1 1 2 3 4 >=5 |
| F1.7 | | Last week, how many DAYS did you eat brown or lal rice?  *(Skip question I1.7a if response is 0)* | | | 0 1 2 3 4 5 6 7 |
| F1.7a | | How many PORTIONS did you eat per day?  **One portion size means half bowl of rice (cooked)** | | | <1 1 2 3 4 >=5 |
| F1.8 | | Last week, how many DAYS did you eat noodles/pasta?  *(Skip question I1.8a, if response is 0)* | | | 0 1 2 3 4 5 6 7 |
| F1.8a | | How many PORTIONS did you eat per day?  **One portion size means half bowl (cooked)** | | | <1 1 2 3 4 >=5 |
| F1.9 | | Last week, how many DAYS did you eat cereal (cooked or ready-to-eat dry cereal) for example: daliya, suji, oats, corn flakes?  *(Skip question I1.9a, if response is 0)* | | | 0 1 2 3 4 5 6 7 |
| F1.9a | | How many PORTIONS did you eat per day?  **One portion size means:**  **Half (1/2) bowl (cooked) cereal**  **3/4 bowl ready-to eat dry cereal** | | | <1 1 2 3 4 >=5 |
| F1.10 | | Last week, how many DAYS did you eat potato in any form, for example, in curry, rice, with roti, boiled, or fried?  *(Skip question I1.10a, if response is 0)* | | | 0 1 2 3 4 5 6 7 |
| F1.10a | | How many PORTIONS did you eat per day?  **One portion size means 1 medium potato** | | | <1 1 2 3 4 >=5 |
| F1.11 | | Last week, how many DAYS did you eat corn (bhutta, popcorn, sweet corn)?  *(Skip question I1.11a, if response is 0)* | | | 0 1 2 3 4 5 6 7 |
| F1.11a | | How many PORTIONS did you eat per day?  **One portion size means:**  **1 medium corn**  **2 bowl of popcorns** | | | <1 1 2 3 4 >=5 |
| F1.12 | | In case of food item consumed other than mentioned ___________  Last week, how many DAYS did you eat that food item? | | | 0 1 2 3 4 5 6 7 |
| F1.12a | | How many PORTIONS did you eat per day? | | | <1 1 2 3 4 >=5 |

| **Section F Part 2: Vegetables** | | |
| --- | --- | --- |
| F2.1 | Last week, how many DAYS did you eat cooked vegetables (boiled/ stir fried or cooked another way) for example: lady finger, bitter gourd, bottle gourd, cauliflower, sponge gourd, tinday, spinach, brinjal, peas?  *(Skip question I2.1a, if response is 0)* | 0 1 2 3 4 5 6 7 |
| F2.1a | How many PORTIONS did you eat per day?  **One portion size means: half bowl of cooked vegetable.** | <1 1 2 3 4 >=5 |
| F2.2 | Last week, how many DAYS did you eat raw vegetables (for example: Lettuce, Tomatoes, cucumber, beetroot, carrot, radish)?  *(Skip question I2.2a if response is 0)* | 0 1 2 3 4 5 6 7 |
| F2.2a | How many PORTIONS did you eat per day?  **One portion size means: one bowl of raw vegetables.** | <1 1 2 3 4 >=5 |
| F2.3 | In case of food item consumed other than mentioned ___________  Last week, how many DAYS did you eat that food item? | 0 1 2 3 4 5 6 7 |
| F2.3a | How many PORTIONS did you eat per DAY? | <1 1 2 3 4 >=5 |

| **Section F Part 3: Fruits** | | |
| --- | --- | --- |
| F3.1 | Last week, how many DAYS did you eat fruits (for example: Apples, pears, mangoes, peaches, bananas, oranges or other citrus fruits)?  *(Skip question I3.1a, if response is 0)* | 0 1 2 3 4 5 6 7 |
| F3.1a | How many PORTIONS did you eat per day?  **One portion size means:**  **Medium size one fruit**  **Large size half fruit.** | <1 1 2 3 4 >=5 |
| F3.2 | Last week, how many DAYS did you eat any other fruit (for example: Strawberries, cherries, grapes, papaya, melons, chico or apricot.)?  *(Skip question I3.2a, if response is 0)* | 0 1 2 3 4 5 6 7 |
| F3.2a | How many PORTIONS did you eat per day?  **One portion size means:**  **A small cup of strawberries, cherries, grapes, papaya, melons**  **2 small fruits such as Chico or apricot.** | <1 1 2 3 4 >=5 |
| F3.3 | Last week, how many DAYS did you eat fruit in some other form, such as canned or tinned fruits?  *(Skip question I3.3a, if response is 0)* | 0 1 2 3 4 5 6 7 |
| F3.3a | How many PORTIONS did you eat per day?  **One portion size means half bowl.** | <1 1 2 3 4 >=5 |
| F3.4 | Last week, how many DAYS did you eat any dried fruit, for example: unsalted and unfried walnut (without shell), almond, peanut, cashew nuts, dates, or figs?  *(Skip question I3.4a, if response is 0)* | 0 1 2 3 4 5 6 7 |
| F3.4a | How many PORTIONS did you eat per day?  **One portion size means quarter bowl or 10-12 pieces.** | <1 1 2 3 4 >=5 |
| F3.5 | Last week, how many DAYS did you drink fresh fruit juice? (for example: Orange juice, apple juice, coconut water, pomegranate juice, mango juice or sugar cane juice etc., without any added sugars)  *(Skip question I3.5a, if response is 0)* | 0 1 2 3 4 5 6 7 |
| F3.5a | How many GLASSES did you drink per day?  **(one glass is equal to 250ml)** | <1 1 2 3 4 >=5 |
| F3.6 | Last week, how many DAYS did you drink fruit milkshakes?  *(Skip question I3.6a if response is 0)* | 0 1 2 3 4 5 6 7 |
| F3.6a | How many GLASSES did you drink per day?  **(one glass is equal to 250ml)** | <1 1 2 3 4 >=5 |
| F3.7 | In case of food item consumed other than mentioned ___________  Last week, how many DAYS did you eat that food item? | 0 1 2 3 4 5 6 7 |
| F3.7a | How many PORTIONS did you eat per day? | <1 1 2 3 4 >=5 |

| **Section F Part 4: Meat and meat substitutes from animal and plant sources** | | |
| --- | --- | --- |
| **Red meat: Mutton and beef** | | |
| F4.1 | Last week, how many DAYS did you eat red meat such as mutton and beef?  *(Skip question I4.1a, if response is 0)* | 0 1 2 3 4 5 6 7 |
| F4.1a | How many PORTIONS did you eat per day?  **One portion size means:**  **Mutton: 2-3 small pieces with bone**  **Beef: palm of the hand** | <1 1 2 3 4 >=5 |
| **Lean meat: Chicken** | | |
| F4.2 | Last week, how many DAYS did you eat chicken?  *(Skip question I4.2a, if response is 0)* | 0 1 2 3 4 5 6 7 |
| F4.2a | How many PORTIONS did you eat per day?  **One portion size means half boneless piece or 1 piece with bone** | <1 1 2 3 4 >=5 |
| **Lean meat: Fish and fish products** | | |
| F4.3 | Last week, how many DAYS did you eat cooked or fried fish or fish products?  *(Skip question I4.3a, if response is 0)* | 0 1 2 3 4 5 6 7 |
| F4.3a | How many PORTIONS did you eat per day?  **One portion size means the size of the palm / one fillet** | <1 1 2 3 4 >=5 |
| F4.4 | Last week, how many DAYS did you eat cooked or fried shrimp, prawns, crabs or lobster?  *(Skip question I4.4a, if response is 0)* | 0 1 2 3 4 5 6 7 |
| F4.4a | How many PORTIONS did you eat per day?  **One portion size means:**  **Prawns: quarter bowl (for small size) or 3-4 (big size prawns)** | <1 1 2 3 4 >=5 |
| **Organ meat** | | |
| F4.5 | Last week, how many DAYS did you eat organ meat such as brain or kidney?  (*Skip question I4.5a, if response is 0)* | 0 1 2 3 4 5 6 7 |
| F4.5a | How many PORTIONS did you eat per day?  **One portion size means the size of the palm** | <1 1 2 3 4 >=5 |
| **Liver or liver products** | | |
| F4.6 | Last week, how many DAYS did you eat liver or liver products?  (*Skip question I4.6a, if response is 0)* | 0 1 2 3 4 5 6 7 |
| F4.6a | How many PORTIONS did you eat per day?  **One portion size means the size of the palm** | <1 1 2 3 4 >=5 |
| **Egg** | | |
| F4.7 | Last week, how many DAYS did you eat egg (boiled, omelette, scrambled)  *(Skip question I4.7a, if response is 0)* | 0 1 2 3 4 5 6 7 |
| F4.7a | How many PORTIONS did you eat per day?  **One portion size means 1 egg** | <1 1 2 3 4 >=5 |
| **Protein from plant source** | | |
| F4.8 | Last week, how many DAYS did you eat lentils, pulses, beans and chickpeas?  *(Skip question I4.8a, if response is 0)* | 0 1 2 3 4 5 6 7 |
| F4.8a | How many PORTIONS did you eat per day?  **One portion size means half a bowl (cooked)** | <1 1 2 3 4 >=5 |
| F4.9 | In case of food item consumed other than mentioned ___________  Last week, how many DAYS did you eat that food item? | 0 1 2 3 4 5 6 7 |
| F4.9a | How many PORTIONS did you eat per day? | <1 1 2 3 4 >=5 |

| **Section F Part 5: Milk and milk products** | | |
| --- | --- | --- |
| F5.1 | Last week, how many DAYS did you have milk?  (for example: milk, milkshakes)  *(Skip question I5.1a, if response is 0)* | 0 1 2 3 4 5 6 7 |
| F5.1a | How many PORTIONS did you have per day?  **One portion size means:**  **Milk: 1 cup (250 ml)** | <1 1 2 3 4 >=5 |
| F5.2 | Last week, how many DAYS did you have milk products?  (for example: yoghurt, cheese, lassi)  *(Skip question I5.2a, if response is 0)* | 0 1 2 3 4 5 6 7 |
| F5.2a | How many PORTIONS did you have per day?  **One portion size means:**  **Yogurt: 1 cup yogurt (200 ml)**  **Lassi: one and a half glasses (thick lassi) 375 ml**  **Cottage cheese: size of a matchbox**  **Cheese: 1 slice** | <1 1 2 3 4 >=5 |
| F5.3 | In case of food item consumed other than mentioned ___________  Last week, how many DAYS did you have that food item? | 0 1 2 3 4 5 6 7 |
| F5.3a | How many PORTIONS did you have per day? | <1 1 2 3 4 >=5 |

| **Section F Part 6: oil and fats** | | | |
| --- | --- | --- | --- |
| F6.1 | What do you mostly use for cooking?  *(Skip question I6.2 and I6.3 if answer is butter, margarine or ghee)* | | 1. Oil 2. Butter 3. Margarine 4. Ghee |
| F6.2 | Which type of oil do you use for cooking? | | 1. Mustard oil 2. Sun flower oil 3. Soya bean oil 4. Coconut oil 5. Olive oil 6. Canola oil 7. Others (specify) _________ |
| F6.3 | How much oil is used within a month? | | _________ liter |
| **Section F Part 7: Savoury snacks** | | | |
| F7.1 | Last week, how many DAYS did you eat savoury snacks? (for example: crisps, fries, salted nuts, dried soups, ready-to-eat noodles, pakoray, samosay, spring rolls, sauces, pickles or chutneys).  *(Skip question I7.1a, if response is 0)* | 0 1 2 3 4 5 6 7 | |
| F7.1a | How many PORTIONS did you eat per DAY?  **One portion size means 1 item/piece of size of a matchbox** | <1 1 2 3 4 >=5 | |
| **Sweet snacks or desserts** | | | |
| F7.2 | Last week, how many DAYS did you eat sweet snacks or desserts? (for example: slice of cake, pastry, cookies, sweets or methai, chocolates, ice cream, savayia, jam, jellies, marmalade, rabrri, honey)  *(Skip question I7.2a, if response is 0)* | 0 1 2 3 4 5 6 7 | |
| F7.2a | How many PORTIONS did you eat per day?  **One portion size means 1 item/piece of size of a matchbox** | <1 1 2 3 4 >=5 | |
| F7.3 | Last week, how many DAYS did you drink packaged fruit juice? (for example: Orange juice, apple juice, coconut water, pomegranate juice, mango juice or sugar cane juice etc.)  *(Skip question I7.3a, if response is 0)* | 0 1 2 3 4 5 6 7 | |
| F7.3a | How many GLASSES did you drink per day?  **(one glass is equal to 250ml)** | <1 1 2 3 4 >=5 | |
| **Section F Part 7: Ready-made meals and fast food** | | | |
| F7.4 | Last week, how many DAYS did you eat ready meals and fast food? (for example: takeaway food, burger, pizza, kebabs, fish and chips, frozen food etc.)  *(Skip question I7.4a, if response is 0)* | | 0 1 2 3 4 5 6 7 |
| F7.4a | How many PORTIONS did you eat per day?  **One portion size means 1 item/piece of size of a matchbox** | | <1 1 2 3 4 >=5 |
| F7.5 | In case of food item consumed other than mentioned ___________  Last week, how many DAYS did you eat that food item? | | 0 1 2 3 4 5 6 7 |
| F7.5a | How many PORTIONS did you eat per day? | | <1 1 2 3 4 >=5 |

| **Section F Part 8: Water Intake** | | |
| --- | --- | --- |
| F8.1 | On average, how many GLASSES of water do you take daily? | ___________ Glasses |

| **Section G: Screening for Lifestyle Habits** | | |
| --- | --- | --- |
| **Tea, Coffee and Carbonated Beverages** | | |
| G1 | Last week, how many DAYS did you drink tea? (Black tea or tea with milk).  *(Skip question J1a, if response is 0)* | 0 1 2 3 4 5 6 7 |
| G1a | How many CUPS did you have per day?  **1 cup= 250 ml** | <1 1 2 3 4 >=5 |
| G2 | Last week, how many DAYS did you drink coffee?  *(Skip question J2a, if response is 0)* | 0 1 2 3 4 5 6 7 |
| G2a | How many CUPS did you have per day?  **1 cup= 250 ml** | <1 1 2 3 4 >=5 |
| G3 | Last week, how many DAYS did you drink carbonated beverages/soda or diet soda?  *(Skip question J4a, if response is 0)* | 0 1 2 3 4 5 6 7 |
| G3a | How many GLASSES/BOTTLES did you have per day?  **1 bottle= 250 ml**  **I glass= 250 ml** | <1 1 2 3 4 >=5 |
| G4 | Do you add or sprinkle additional salt to the food you eat?  *(Skip Question J5a if response is No)* | 1. Yes 2. No |
| G4a | How frequently do you do that in a week? | 1. Always (7 days) 2. Often (4-6 days) 3. Sometimes (2-3 days) 4. Rarely (once a week) |

Bottom of Form
